# Indirect ELISA Using Multi-Antigenic Dominants of VP1, VP2, and VP3 Recombinant Protein to Detect Antibodies Against Senecavirus A in Pigs

**DOI:** 10.3390/vetsci13010090

**Published:** 2026-01-15

**Authors:** Zenglin Wang, Dexin Li, Yufang Li, Yunjing Zhang, Junhua Deng, Liying Hao, Kegong Tian, Xiangdong Li

**Affiliations:** 1 Jiangsu Co-Innovation Center for Prevention and Control of Important Animal Infectious Diseases and Zoonoses, College of Veterinary Medicine, Yangzhou University, Yangzhou 225009, China; 18763641225@163.com (Z.W.); lidexin0123@163.com (D.L.); 2 Luoyang Putai Biotechnology Co., Ltd., Luoyang 471003, China; liyufang123@126.com (Y.L.); deng.junhua@sslab.com.cn (J.D.); haoly0717@163.com (L.H.); 3 National Research Center for Veterinary Medicine, Luoyang 471003, China; zhangyunjing@pulike.com.cn; 4 Joint International Research Laboratory of Agriculture and Agri-Product Safety, The Ministry of Education of China, Yangzhou University, Yangzhou 225009, China

**Keywords:** Senecavirus A, immunodominance, indirect ELISA, serosurvey

## Abstract

Senecavirus A (SVA) is an emerging pathogen that poses a significant threat to the global swine industry. With the advent of SVA vaccines, there is a growing need to develop serological diagnostic methods for evaluating vaccine-induced immunity. This study successfully established an indirect enzyme-linked immunosorbent assay (iELISA) through heterologous expression of a novel VP2-VP3-VP1 tandem recombinant protein in *Escherichia coli* (*E. coli*), which was constructed by integrating B-cell epitopes from VP1, VP2, and VP3. Comparative analysis using indirect ELISA revealed that the tandem recombinant VP2-VP3-VP1 protein and VP2 exhibited superior immunoreactivity. Consequently, the iELISAs for the tandem protein and VP2 were selected for further validation. Following optimization, the cut-off for the rVP2-VP3-VP1 iELISA was set at a sample-to-positive (S/P) ratio ≥ 0.60, while that for the rVP2 iELISA was set at ≥0.53. Analysis of kinetic sera from inactivated vaccine-immunized pigs showed that the rVP2-VP3-VP1 iELISA detected seroconversion synchronously with neutralizing antibodies, earlier than anti-VP2 antibodies. Finally, a serological survey for SVA was conducted in parts of mainland China from 2023 to 2024, with the rVP2-VP3-VP1 iELISA revealing an overall seroprevalence of 20.8%. These results indicate that the established detection method can be effectively used to evaluate SVA immunity and for epidemic surveillance.

## Simple Summary

Senecavirus A (SVA) is defined as an emerging viral agent responsible for inducing vesicular lesions in swine. The recent development of SVA vaccines has created a need for reliable methods to evaluate post-vaccination immune responses for diagnostic and monitoring purposes. Here, we developed a serological assay based on a designed tandem antigen, VP2-VP3-VP1, as its core component. The assay demonstrated high specificity and sensitivity, confirming its suitability for assessing immune responses following vaccination. When applied to a panel of more than 3800 porcine serum samples, the assay demonstrated high reliability and confirmed its utility for large-scale serological surveillance. In summary, this newly developed serological tool is valuable for the reliable monitoring of vaccination efficacy and outbreak surveillance.

## 1. Introduction

Senecavirus A (SVA) is a non-enveloped, single-stranded RNA virus in the genus *Senecavirus*, family *Picornaviridae*, that primarily causes vesicular disease in swine [1,2]. It was first identified in the United States in 2002, initially regarded as a contaminant in cell culture, and subsequently isolated and named SVV-001 [3]. By 2015, cases of SVA infection began to be reported in several countries [4], including the United States [5], Chile [6], Brazil [7], Colombia [8], and Vietnam [9]. In 2015, the first case of SVA infection was reported in Guangdong Province, China, and the virus subsequently spread to multiple other provinces within the country [10,11,12]. These findings indicate that SVA not only has a wide geographical distribution but also spreads rapidly. The clinical signs induced by SVA infection are highly similar to those caused by pathogens such as the Foot-and-mouth disease virus (FMDV), Vesicular stomatitis virus (VSV), and Swine Vesicular Disease virus (SVDV). This similarity complicates the clinical diagnosis of SVA [13].

SVA is characterized by an icosahedral capsid and a genome of approximately 7.2 kilobases [3]. Its genome features a VPg protein covalently linked to the 5′ end, encoding the four structural proteins (VP4, VP2, VP3, and VP1), while the 3′ terminus contains a poly(A) tail and encodes seven non-structural proteins (2A, 2B, 2C, 3A, 3B, 3C, and 3D) [3,4]. Of these structural proteins, VP1–VP3 form the exterior of the capsid and are the principal targets of neutralizing antibodies, in contrast to the internally located VP4 [4]. Conversely, non-structural proteins are not part of the virion but are indispensable for processes critical to the viral life cycle, including genome replication and host interaction [14]. Within picornaviruses, the VP1 protein, located on the outermost surface and being highly immunogenic [15], has been utilized in various serological assays for antibody detection. In SVA structural protein-based ELISA for swine, VP2 antigen was substantially more immunogenic than VP1 and VP3 in the population as a whole, although occasional pigs were observed with high antibody levels to one or both of VP1 and VP3 [16].

To date, several serological diagnostic methods for SVA are available, including the virus neutralization test (VNT) [17], indirect immunofluorescence assay (IFA) [16,17], and indirect or competitive ELISAs [4]. According to a previous report, VNT is considered the gold standard for detecting SVA antibodies in sera [17]. However, VNT requires live SVA and specific laboratory facilities, making it technically complex and time-consuming to perform. As a serological method, ELISA is widely used in clinical testing due to its rapidity and simplicity [18]. With the ongoing development of SVA inactivated and subunit vaccines [19,20,21], it is imperative to establish a simple, accurate, and reliable ELISA method for evaluating vaccine immunogenicity.

In this study, we expressed the recombinant proteins rVP1, rVP2, and rVP3, as well as a tandem protein (rVP2-VP3-VP1) incorporating their key immunodominant regions, in *E. coli*. These four antigens were used to develop indirect ELISAs. Following immunoreactivity screening, the resulting rVP2-VP3-VP1-coated indirect ELISA demonstrated higher sensitivity and specificity than single-antigen assays, confirming its utility as a dependable assay for SVA serology. Finally, the established method was applied to detect 3851 clinical serum samples collected from different provinces and time points to analyze seroprevalence. Our findings offer valuable insights into SVA antibody detection and contribute to the understanding of its seroprevalence.

## 2. Materials and Methods

### 2.1. Virus Strains, Antibodies, and Sera

The SVA CH-HNXC strain was prepared in our previous study [22]. IBRS-2 cells were maintained in Dulbecco’s Modified Eagle Medium (DMEM; Gibco, Waltham, MA, USA) supplemented with 8% fetal bovine serum (FBS; Biological Industries, Kibbutz Beit Haemek, Israel) and incubated at 37 °C with 5% CO_2_.

The secondary antibody employed was horseradish peroxidase (HRP)-conjugated goat anti-pig IgG (H + L) (Abcam, Waltham, MA, USA).

A total of 200 serum samples were sourced from the National Research Center for Veterinary Medicine, comprising 100 VNT SVA-positive and 100 VNT SVA-negative sera and additional immune sera against the inactivated SVA vaccine, prepared in our previous study [22].

For specificity assessment, reference positive sera against multiple swine pathogens were included: Porcine Reproductive and Respiratory Syndrome Virus (PRRSV), Porcine Circovirus Type 2 (PCV2), Foot-and-mouth disease virus (FMDV) serotypes A and O, Pseudorabies Virus (PRV), Porcine Circovirus Type 3 (PCV3), and Classical Swine Fever Virus (CSFV) positive serum were provided from Beijing Sino-science Gene Technology Co., Ltd. (Beijing, China), while African Swine Fever Virus (ASFV) antibody-positive sera were acquired from the China Institute of Veterinary Drug Control (Beijing, China). The serostatus of FMDV-positive samples was verified in our previous study [22].

To conduct a comprehensive serosurvey, we collected 3851 field-collected swine serum samples from 216 farms throughout the China. These samples, provided by Luoyang Putai Biotechnology Co., Ltd., Luoyang, China, were analyzed to investigate the seroprevalence of SVA.

### 2.2. Screening of Dominant Epitopes and Plasmid Construction

**Figure 1:**
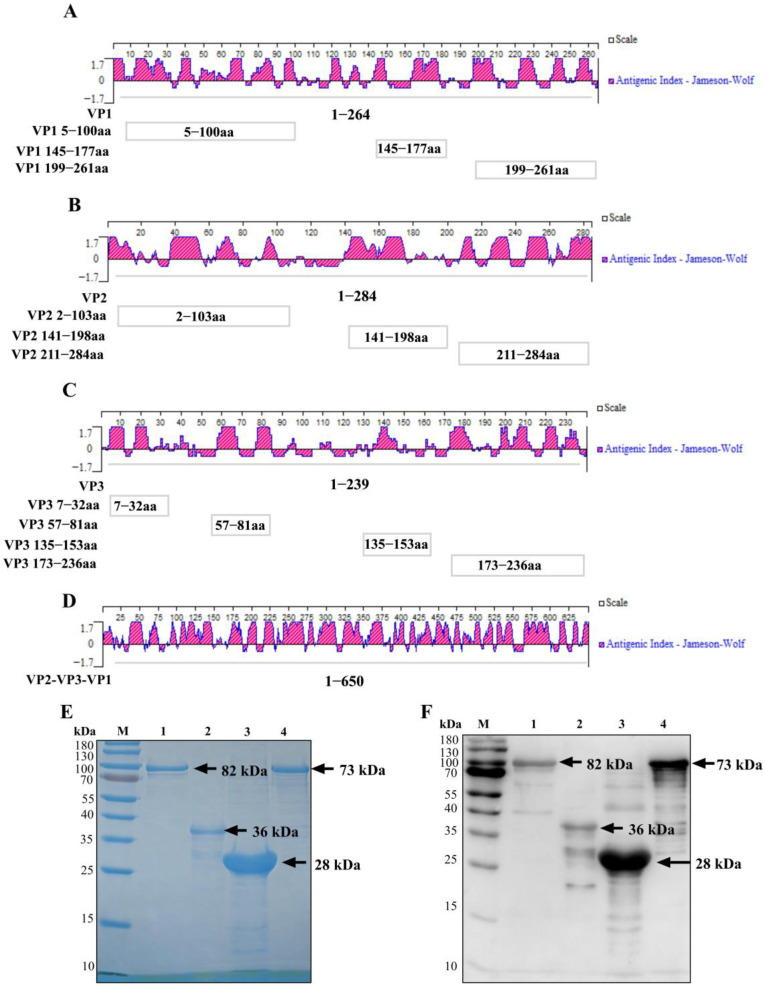
Generation, Purification, and Characterization of SVA Target Proteins. (**A**–**C**) Antigenicity index of VP1 (**A**), VP2 (**B**), and VP3 (**C**) proteins using the Jameson–Wolf algorithm. Epitope regions are highlighted: VP1 (aa 5–100, 145–177, and 199–261), VP2 (aa2–103, 141–198, and 211–284), and VP3 (aa 7–32, 57–81, 135–153, and 173–236). (**D**) Antigenic index of the VP2-VP3-VP1 tandem protein. (**E**) SDS-PAGE analysis of the expressed proteins, showing their size and purity. (**F**) Western blot confirming the reactivity of the recombinant proteins with SVA-positive swine serum. Lanes: M, protein molecular weight marker; 1, VP1 (~82 kDa); 2, VP2 (~36 kDa); 3, VP3 (~28 kDa); 4, VP2-VP3-VP1 (~73 kDa). The lane order is consistent between (**E**) and (**F**), (the original western blot pictures can be found in Figure S1).

Bioinformatic analysis was performed to identify potential immunodominant regions in the SVA structural proteins VP1, VP2, and VP3. The antigenic indices were analyzed using DNAStar Protean (version 7.1.0), and linear epitopes were predicted using the BepiPred Linear Epitope Prediction tool (IEDB Analysis Resource) from the IEDB Analysis Resource. Immunodominant epitopes were identified through a combination of these computational predictions and previously documented epitope information from established literature. The incorporated epitopes were ^21^GELAAP^26^ for VP1 [23]; ^12^DRVITQT^18^, ^71^WTKAVK^76^, ^98^GGAFTA^103^, ^150^KSLQELN^156^, and ^248^YKEGAT^253^ for VP2 [23], along with ^156^NEEQWV^161^ and ^262^VRPTSPYFN^270^ [24], and ^153^QELNEE^158^ [25]; and ^192^GWFSLHKLTK^201^ for VP3 [26]. The final selected epitopic regions (Figure 1A–C) comprised three segments in VP1 (aa 5–100, 145–177, and 199–261), three in VP2 (aa 2–103, 141–198, and 211–284), and four in VP3 (aa 7–32, 57–81, 135–153, and 173–236).

A recombinant tandem gene was engineered by connecting the selected epitopes from VP1, VP2, and VP3 with flexible glycine-serine linkers (GGGGS)_2_, generating the VP2-VP3-VP1 construct (73 kDa). This customized gene sequence was optimized for *Escherichia coli* codon usage, chemically synthesized and then inserted into the pCold-I expression vector (GenScript, Nanjing, China) through *Nde*I/*BamH*I restriction sites. All primers are listed in Table 1.

### 2.3. Protein Expression and Purification

Following transformation into *E. coli* BL21(DE3) host cells, recombinant clones were selected and cultured at 37 °C in LB medium comprising the respective antibiotics (0.1 mg/mL ampicillin for pCold-derived constructs; 0.05 mg/mL kanamycin for pET-28a), with agitation at 220 rpm. When the OD_600_ reached 0.4–0.6, induction was initiated by adding IPTG (Solarbio, Beijing, China). Constructs based on pCold vectors (VP1, VP3, and VP2-VP3-VP1) were induced with 0.1 mM IPTG at 16 °C for 16–20 h, whereas the pET-28a-VP2 construct was induced with 1 mM IPTG at 37 °C for 5–6 h.

Post-induction cells were collected by centrifugation (8000 rpm, 5 min) and subjected to ultrasonic disruption in an imidazole-containing buffer (10 mM) on ice. Lysates were clarified by centrifugation (4000 rpm, 10 min, 4 °C) to yield soluble (supernatant) and insoluble (pellet) fractions, which were subsequently analyzed by SDS-PAGE (Tanon; Shanghai, China) to evaluate protein expression profiles.

Proteins were affinity-purified employing Ni-NTA resin (GenScript, Piscataway, NJ, USA) following the manufacturer’s instructions. For soluble proteins, the resin was first subjected to pre-equilibration and washing with washing buffer (50 mM NaH_2_PO_4_, 300 mM NaCl, 250 mM imidazole, pH 8.0) at room temperature, followed by elution (250 mM imidazole) to recover the target protein. Conversely, insoluble proteins were first dissolved in a denaturing buffer (50 mM Tris, 500 mM NaCl, 8 M urea, pH 7.5), bound to the resin, washed with a denaturing wash buffer (50 mM Tris, 500 mM NaCl, 10–20 mM imidazole, 8 M urea, pH 7.5), and finally eluted using an elution solution for denaturing conditions, supplemented with 250 mM imidazole and 8 M urea.

### 2.4. SDS-PAGE and Western Blotting Analysis

After SDS-PAGE separation, proteins were electrotransferred onto a PVDF membrane (Millipore, Darmstadt, Germany). The procedure subsequently included blocking the PVDF membrane using 5% BSA (Solarbio, Beijing, China) for 2 h at room temperature. Following incubation with the primary antibody (SVA antibody-positive pig serum, 1:1000) for 2 h, the membrane was washed five times with TBST (Tris-buffered saline with 0.05% Tween-20; Solarbio, Beijing, China). The membrane was then treated with HRP-conjugated goat anti-pig IgG (1:10,000) for 1 h at room temperature. Following the final wash, target proteins on the PVDF membrane were detected using a chemiluminescence imaging system.

### 2.5. Virus Neutralization Test (VNT)

Neutralizing antibody titers in porcine sera were quantified using a fixed-virus, diluted-serum format. Sera were first subjected to heat inactivation (56 °C, 30 min). Subsequently, two-fold serial dilutions of the inactivated sera were prepared across a 96-well plate. Each sample dilution was combined with an equal volume of SVA suspension containing 200 50% tissue culture infective dose (TCID_50_)/50 μL. The virus–serum complexes were incubated for 1 h at 37 °C during the neutralization period.

Following neutralization, IBRS-2 cells were added to each well at 50 μL. Plates were maintained at 37 °C in a 5% CO_2_-humidified environment and monitored daily for three days to assess the cytopathic effect (CPE).

Control wells included uninfected cell controls, serum toxicity controls, and virus controls. The endpoint neutralization titer was established by identifying the maximum serum dilution that protected at least 50% of the cultured cells from CPE and calculating its reciprocal.

### 2.6. Development of the Indirect ELISA

Antigen coating was performed by immobilizing purified proteins onto ELISA plates at varying concentrations (100–500 ng/well), followed by overnight incubation at 4 °C. Subsequent blocking was performed using 2.5% BSA for 24 h at 4 °C. The prepared plates were then air-dried (20–25 °C, 16–20 h) and stored at 2–8 °C until further use.

A checkerboard assay was used to optimize reaction parameters systematically. Each assay point was measured with three replicate wells. Preliminary assessment under standardized conditions (37 °C, 30 min for serum and secondary antibody incubation; 15 min 3,3′,5,5′-tetramethylbenzidine (TMB) development) established baseline performance for antigen coating concentration, serum dilution (1:25–1:100), and secondary antibody dilution (1:5000–1:40,000). Further refinement focused on evaluating the incubation kinetics for both serum and secondary antibody (15–60 min).

Parameter selection was guided by maximizing the positive-to-negative (P/N) ratio to ensure optimal signal-to-background differentiation. The finalized protocol was verified through triplicate testing with five SVA-positive and three negative reference sera.

All buffers used in the ELISA procedure were supplied by Luoyang Putai Biotechnology Co., Ltd. The buffers and their main components are listed below: Coating buffer (0.05 M Carbonate–Bicarbonate Buffer, pH 9.6), Blocking buffer (2.5% BSA in PBS), Washing buffer (Phosphate-Buffered Saline (PBS) with 0.05% Tween-20), Serum and antibody dilution buffer (PBST containing 0.5% BSA).

### 2.7. Cut-Off Values of the Indirect ELISA

We established the cut-off values for the VP2 and VP2-VP3-VP1-based iELISA assays using 200 well-defined porcine serum specimens with confirmed VNT status (100 positive, 100 negative). Following ELISA testing and OD_450_ measurement, diagnostic thresholds were determined separately for each antigen.

The sample-to-positive (S/P) ratio for each sample was derived from the optical density (OD_450_) values as follows: S/P = (OD_450_ Sample − OD_450_ Negative control)/(OD_450_ Positive control − OD_450_ Negative control). Diagnostic performance was subsequently evaluated through receiver operating characteristic (ROC) curve analysis of S/P ratios using GraphPad Prism 8.0. For each antigen, the optimal diagnostic threshold was identified by maximizing Youden′s index, with corresponding sensitivity and specificity calculated at this optimal Cut-off value.

### 2.8. Assay Performance Evaluation: Specificity, Sensitivity and Precision

The specificity of the assay was determined by testing it against sera confirmed to be positive for other prevalent pig pathogens, PRV, PCV2, PRRSV, PCV3, ASFV, and CSFV. The panel included twenty positive specimens for FMDV serotypes O and A, while three positive samples were examined for each of the remaining pathogens. For each serum sample, testing was performed with three replicate wells.

To determine the analytical sensitivity, a high-titer reference serum positive for SVA antibodies was subjected to cover a range from 1:2 to 1:128. The endpoint titer was defined as the maximum dilution factor that generated a positive reading in the assay system.

To assess diagnostic sensitivity, serum samples collected from pigs following administration of the inactivated vaccine were analyzed. The ELISA performance was benchmarked against virus-neutralization titers as the reference standard.

Assay precision was assessed using three positive sera of three different strengths. Eight replicate measurements of each sample within a single run characterized the intra-assay precision. Data from eight replicate tests per sample on each of three separate plates served to evaluate the inter-assay reproducibility.

### 2.9. SVA Serological Survey

The established antibody detection assay was applied to analyze 3851 pig serum samples collected from 48 cities across 20 provinces between 2023 and 2024. The analysis aimed to determine SVA antibody seroprevalence, seasonal distribution, and association with pig age.

### 2.10. Data Analysis

All statistical analyses and graph generation were performed using GraphPad Prism 8.0 (GraphPad Software, San Diego, CA, USA). Statistical analyses were performed using Student’s *t*-tests. A threshold of *p* < 0.05 was applied for statistical significance. Significance levels are denoted as follows: * *p* < 0.05, ** *p* < 0.01, *** *p* < 0.001; ns, not significant.

## 3. Results

### 3.1. Screening of Dominant Epitopes and Expression and Purification of Recombinant Proteins

Following the identification of immunodominant epitopes through bioinformatic analysis, expression plasmids encoding the tandem antigen were engineered as outlined in Section 2.2, with the overall construct design illustrated in Figure 1A–D.

Based on their solubility following expression in *E. coli*, the recombinant proteins were purified under appropriate conditions: VP1 (soluble) was purified under native conditions, while VP2, VP3, and the tandem VP2-VP3-VP1 protein (insoluble, from inclusion bodies) were purified under denaturing conditions using Ni-NTA affinity chromatography. Purified proteins showed a single band of the expected size on SDS-PAGE (Figure 1E), and their reactivity was confirmed by Western blot with sera from SVA-infected pigs (Figure 1F).

### 3.2. Development and Evaluation of iELISAs for SVA Proteins

**Figure 2:**
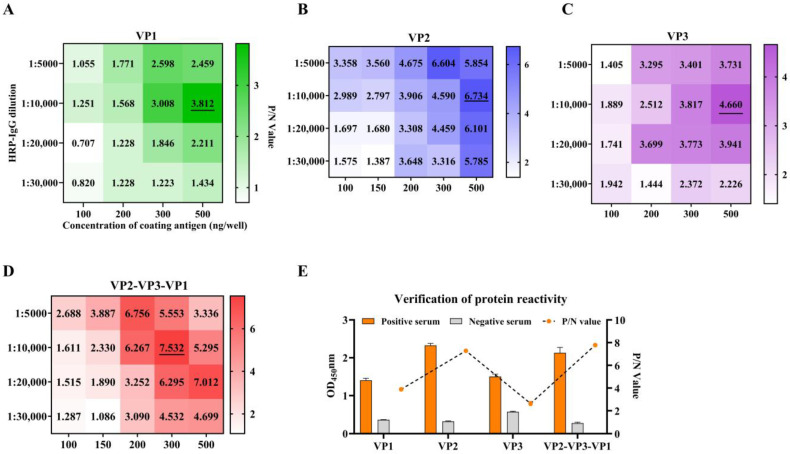
Establishment of iELISAs for SVA target proteins and evaluation of immunoreactivity. (**A**–**D**) Checkerboard titration for determining the antigen coating concentration and secondary antibody dilution for the recombinant VP1, VP2, VP3, and VP2-VP3-VP1 proteins, respectively. Data are presented as heatmaps of the positive-to-negative (P/N) values (generated with GraphPad Prism). For each panel, the optimal condition is marked with an underline. (**E**) Comparison of the optimal immunoreactivity of the single structural proteins and the tandem protein. Orange and gray bars represent the mean OD_450_ values (± SD) of SVA-positive and SVA-negative sera (*n* = 4), respectively, from three independent assays.

A checkerboard assay was used to determine the optimal reaction parameters by maximizing the P/N value. Under a coating concentration of 500 ng/well and a secondary antibody dilution of 1:10,000, the maximum P/N values for VP1, VP2, and VP3 were 3.812, 6.734, and 4.660, respectively. For the VP2-VP3-VP1 tandem protein, a P/N value of 7.532 was achieved at a coating concentration of 300 ng/well with the same antibody dilution (Figure 2A−D).

Comparative analysis established VP2 as the most immunoreactive single structural protein. Notably, the tandem antigen VP2-VP3-VP1 showed enhanced immunoreactivity compared to VP2 alone (Figure 2E). Based on these findings, both VP2 and the VP2-VP3-VP1 tandem protein were advanced for comprehensive validation of their specificity, sensitivity, and repeatability.

### 3.3. Assay Optimization and Establishment of Cut-Off Values

**Figure 3:**
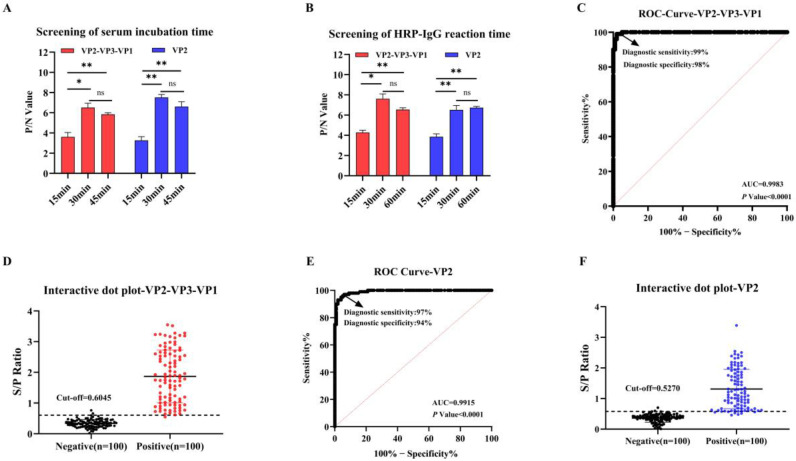
Development and Standardization of the SVA iELISAs: Optimization and Cut-off Establishment. (**A**) Screening of serum incubation time (**B**) Screening of HRP-IgG reaction time (**C**−**F**) Diagnostic performance assessment of the rVP2-VP3-VP1 iELISA and rVP2 iELISA. (**C**,**E**) Analysis of the ROC curves for both iELISAs is presented, with the AUC serving as an indicator of diagnostic accuracy. (**D**,**F**) Dot plots of the sample-to-positive (S/P) ratios for each individual serum sample. The cut-off was defined by maximizing Youden’s index and is represented by a horizontal dashed line in the figure. Student’s *t*-test evaluated differences, ** *p* < 0.01, * *p* < 0.05; ns, not significant.

To optimize the assay procedures, we next optimized the incubation and reaction times for serum and the HRP-IgG separately for both the VP2 and tandem protein (VP2-VP3-VP1) iELISAs. (Figure 3A,B). Comparative analysis revealed comparable performance between 30 and 45 min serum incubation periods (Figure 3A) and between 30 and 60 min secondary antibody incubation intervals (Figure 3B). The 30 min duration was consequently adopted for both procedural steps to maximize operational efficiency.

Diagnostic sensitivity and specificity were evaluated using 200 samples of known VNT status. As shown in Figure 3C,E, ROC analysis of both the VP2-VP3-VP1 and VP2 iELISAs showed excellent diagnostic accuracy, with respective area under the curve (AUC) of 0.9983 (95% confidence interval (CI): 0.9957–1.000; *p* < 0.0001) and 0.9915 (95% CI: 0.9833–0.9996; *p* < 0.0001). Application of Youden’s index maximization criteria established diagnostic thresholds at S/P ratios of 0.6045 for VP2-VP3-VP1 (Figure 3D) and 0.5270 for VP2 (Figure 3F). To better reflect the practicalities of diagnostic testing. these values were rounded to two decimal places. Consequently, the cut-off value for VP2-VP3-VP1 was set at 0.60, and that for VP2 was set at 0.53. These optimized parameters yielded high diagnostic performance for both assays, with the tandem antigen configuration demonstrating moderately higher diagnostic performance than the single-component VP2 assay.

### 3.4. Repeatability and Reproducibility Assessment

The precision of both iELISAs was evaluated by coefficient of variation (CV) analysis. As shown in Table 2, the tandem rVP2–VP3–VP1 iELISA exhibited intra-assay CVs of 3.4−4.3% and inter-assay CVs of 3.5−5.3%, while the rVP2 iELISA exhibited corresponding ranges of 1.4−2.8% and 3.9−9.7%. The low CV values demonstrate the high precision and robustness of both iELISAs, supporting their suitability for SVA serology.

### 3.5. Specificity, Analytical Sensitivity, and Diagnostic Performance of iELISAs

**Figure 4:**
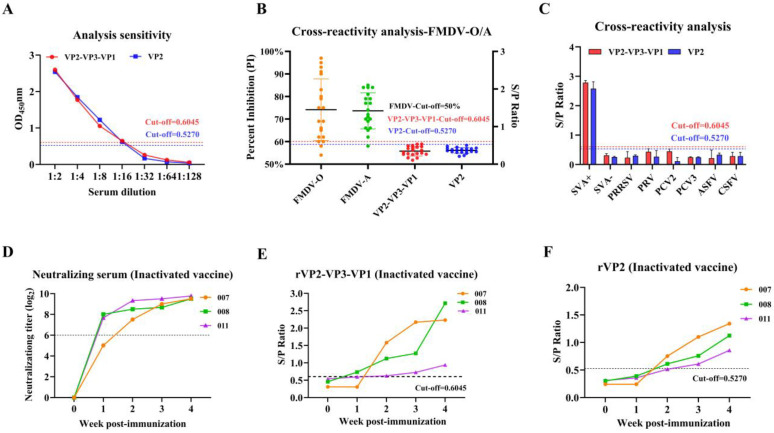
Comparative analysis of sensitivity and specificity between the VP2 and VP2-VP3-VP1 iELISAs. (**A**) A range from 1:2 to 1:128 of a positive sera for SVA served to establish the analytical sensitivity. (**B**) Assessment of reaction with positive serum for FMDV (serotype O/A). The left *Y*-axis represents the FMDV-specific antibody percentage inhibition (obtained from commercial ELISA kits), and the right *Y*-axis shows the S/P values for the SVA antigens. (**C**) Cross-reactivity evaluation of the SVA antigens with sera positive for other prevalent pig pathogens (PCV2, PRV, PCV3, PRRSV, CSFV ASFV,). (**D**) Virus neutralization test (VNT) results for sera from the three inactivated-vaccine immunized pigs. (**E**,**F**) Antibody kinetics detected by the iELISAs based on the (**E**) VP2-VP3-VP1 and (**F**) VP2 proteins, respectively. Across figures (**A**–**F**), the dashed lines are defined by the established cut-offs specific to each assay.

Using two-fold serial dilutions SVA-positive serum to determind the analytical sensitivity of each iELISA. Both the VP2-VP3-VP1-based and the VP2-based iELISAs exhibited identical analytical sensitivity endpoints, with antibodies detectable at a 1:16 dilution in both assays (Figure 4A).

Analytical specificity was assessed by testing sera positive for other swine pathogens. Neither the VP2-VP3-VP1 nor the VP2 antigen showed significant reaction with serum for FMDV (serotype O/A), as confirmed by commercial ELISA kits (Figure 4B). Furthermore, no reactivity was observed against a panel of positive sera for PCV2, PRV, PRRSV, PCV3, CSFV, or ASFV (Figure 4C), validating the high specificity of both iELISAs.

Neutralizing antibody titers served as the benchmark for immune response (Figure 4D). The VP2-VP3-VP1 iELISA demonstrated earlier detection of seroconversion, identifying positive responses in pigs #008 and #011 at 1 week post-immunization (wpi) and in all three pigs by 2 weeks post-immunization (wpi) (Figure 4E). In contrast, the VP2 iELISA showed delayed detection, with seroconversion not observed until 2 wpi and consistent positivity across all animals only by 3 wpi (Figure 4F). These results indicate that the rVP2-VP3-VP1 iELISA offers superior diagnostic sensitivity for monitoring vaccine-induced immune responses compared to the VP2 iELISA.

### 3.6. Seroepidemiological Survey for SVA Monitoring

**Figure 5:**
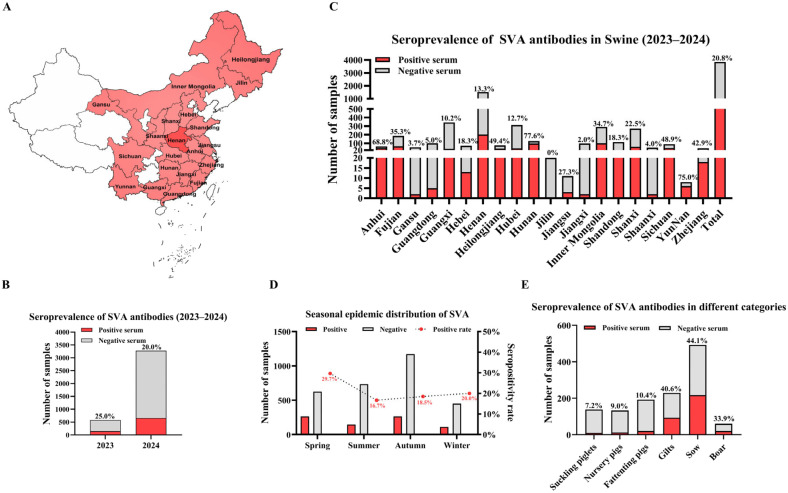
Seroepidemiological of SVA in China (2023−2024). (**A**) Schematic map illustrating the quantity and geographical distribution of the 3851 pig serum samples across Mainland China. Legend: Dark red, >1000 serum samples; light red, 1–1000 serum samples; white, 0 serum samples. (**B**) Annual SVA antibody seroprevalence from 2023 to 2024. (**C**) Provincial SVA antibody seroprevalence across 20 provinces and the overall rate. (**D**) Seasonal dynamics of SVA seroprevalence. (**E**) SVA seroprevalence among different swine groups.

Leveraging the optimized VP2-VP3-VP1 iELISA, a seroepidemiological investigation was undertaken to describe the current SVA situation in China. This study analyzed 3851 porcine serum samples collected during 2023–2024 from 216 farms in 48 cities across 20 provinces (Figure 5A).

Analysis of the surveillance data indicated a declining trend in SVA seropositivity, with the rate falling from 25.0% in 2023 to 20.0% in 2024 (Figure 5B). The overall seropositivity rate across the two years was 20.8% (Figure 5C). At the farm level, 54.6% of the surveyed premises had at least one positive animal (Table 3). Substantial regional variation in SVA infection status was observed. Samples from Henan province showed a positivity rate of 13.3%, while constituting the largest proportion of the collection (39.5%, 1520/3851) (Figure 5C). At the farm level within Henan, a higher proportion (44.1%, 45/102) tested positive for SVA (Table 3).

The epidemic pattern of SVA showed clear seasonal dynamics, with transmission peaking in winter and spring, followed by a marked decline in summer and autumn (Figure 5D). A strong age-associated distribution of seropositivity was also evident. The highest rates were found in sows (44.1%), gilts (40.6%), and boars (33.9%). Conversely, dramatically lower rates were detected in nursery pigs (9.0%), suckling piglets (7.2%), and fattening pigs (10.4%) (Figure 5E). This distinct pattern suggests that maternal antibodies provide critical protection for offspring during early life stages. However, the high seroprevalence within breeding herds underscores a persistent reservoir of infection, posing a significant challenge for inter-farm biosecurity and the safe introduction of new breeding stock.

## 4. Discussion

Senecavirus A (SVA) has emerged as a globally distributed pathogen, inflicting substantial economic losses on the swine industry [3,27]. Outbreaks are characterized by vesicular lesions that progress to ulcerative dermatitis on the snout and coronary bands [5,28]. Critically, these clinical signs are indistinguishable from those of other vesicular diseases, such as FMD, VS, and SVD, complicating field diagnosis [29,30]. The development of various SVA vaccines, including inactivated and epitope-based formulations [19,21,31], underscores the need for a practical evaluation of immune responses. Consequently, the development of reliable and accurate serological assays is paramount for effective SVA detection and surveillance.

Structural proteins on the internal and external surfaces of the viral capsid are key targets for neutralizing antibodies due to their high degree of antigenic conservation. This principle has been successfully applied in FMD diagnostics and vaccine development [32]. Multi-epitope proteins have been successfully used to develop inactivated FMDV vaccines and iELISAs [33]. Previous studies have reported that comparable performance was observed between indirect ELISAs developed using eukaryotically expressed and prokaryotically expressed SVA VP1 proteins [34]. Building on this concept and given the ongoing development of SVA vaccines [19], we designed a novel tandem antigen VP2-VP3-VP1, by integrating immunodominant regions from three SVA structural proteins. This recombinant protein was expressed in a prokaryotic system and purified. A limitation of this study is that the recombinant antigens were used directly after denaturing purification without a refolding step, which may affect epitope exposure. But subsequent experiments confirmed the practical utility of the denatured antigens for serodiagnosis. Comparative immunoreactivity analysis demonstrated that the VP2-VP3-VP1 tandem protein was significantly superior to the individual VP1, VP2, and VP3 proteins. Among the single structural proteins, VP2 exhibited higher reactivity than VP1 and VP3, consistent with previous findings [16]. These results demonstrate that the rVP2-VP3-VP1-based iELISA is highly sensitive, confirming the utility of this multi-antigen approach for SVA serology.

The diagnostic performance was further evaluated using kinetic sera from an inactivated vaccine immunization experiment. Using neutralizing antibody titers as a benchmark, the rVP2-VP3-VP1-based iELISA detected seroconversion to structural proteins markedly earlier than the rVP2-based iELISA. Previous studies have shown that assays targeting individual structural proteins, such as VP1, VP2, and VP3, are effective for monitoring SVA infection in clinical samples [16]. Our findings advance this by demonstrating that the tandem antigen VP2-VP3-VP1 not only maintains this capability but also enhances the sensitivity for early detection of vaccine-induced immune responses, making it particularly valuable for assessing vaccination efficacy.

We subsequently applied the rVP2-VP3-VP1-based iELISA to a large-scale serological survey of 3851 clinical serum samples collected from 20 provinces. It should be noted that the geographical distribution of samples was rough, with the largest subset of samples (39.5%; 1520/3851) contributed by Henan Province, which may affect the accuracy of nationwide infection rate extrapolation. Nevertheless, the assay revealed consistent and robust epidemiological patterns. The data showed a declining trend in SVA seroprevalence from 2023 to 2024 and a seasonal distribution, with higher activity in winter and spring compared to summer and autumn, consistent with previously reported patterns [22,27]. Analysis by growth stage revealed significantly lower seropositivity in suckling and nursery pigs than in older animals, suggesting a protective effect of maternal antibodies. Conversely, the high seropositivity in sows, gilts, and boars indicates that breeding stock is a key reservoir, posing a risk to its management. The consistency of these epidemiological patterns with those obtained from our previous NSP-based assay confirms the reliability of the rVP2-VP3-VP1-based iELISA for future clinical diagnosis and serological monitoring [22].

## 5. Conclusions

In this study, we developed a serological assay for SVA diagnosis based on a multi-epitope tandem antigen that incorporates immunodominant regions from VP1, VP2, and VP3. The resulting rVP2-VP3-VP1-based iELISA enables specific detection of SVA antibodies, facilitating the monitoring of vaccine-induced immune responses. Its capacity to detect seroconversion early further underscores the value of this tandem antigen as a sensitive diagnostic marker. Collectively, our findings validate the rVP2-VP3-VP1-based iELISA as a reliable, high-throughput platform for accurately assessing SVA-specific immunity in pigs, thereby significantly enhancing both vaccine efficacy evaluation and outbreak surveillance.

## Figures and Tables

**Table 1:** Primer sequences used in this study.

Constructs	Nucleotide Sequence (5′-3′)	Vector
VP1	F: atatcgaaggtaggcatatgATGTCCACCGACAACGCCGAGACTG	pCold-TF
	R: ccctcgagggtaccgagctcTTATTGCATCAGCATCTTCTGCTTG	
VP2	F: gcggatccgaattcgagctcATGGATCGAGTCATAACACAAACG	pET-28a
	R: cggccgcaagcttgtcgacgCCCGGTCTTGAAACGGTTACG	
VP3	F: atatcgaaggtaggcatatgATGGGGCCCATTCCCACAGCACCCA	pCold-I
	R: ccctcgagggtaccgagctcTTAGTGGAACACGTAGGAAGGATTA	
VP2-VP3-VP1	^1^/	pCold-I
	/	
pCold-I/pCold-TF	F: GAGCTCGGTACCCTCGAGGG	/
	R: CATATGCCTACCTTCGATATGATGA	
pET-28a	F: CGTCGACAAGCTTGCGGC	/
	R: GAGCTCGAATTCGGATCCG	

Note: ^1^ The symbol “/” indicates that no primer was required.

**Table 2:** Intra- and inter-assay precision of the VP2 and VP2-VP3-VP1 iELISAs.

Number of Samples	Intra-Assay						Inter-Assay					
	VP2-VP3-VP1			VP2			VP2-VP3-VP1			VP2		
	$\bar{X}$	SD	CV	$\bar{X}$	SD	CV	$\bar{X}$	SD	CV	$\bar{X}$	SD	CV
Positive 1	2.509	0.107	4.3%	2.476	0.034	1.4%	2.492	0.087	3.5%	2.355	0.112	3.9%
Positive 2	1.310	0.044	3.4%	1.057	0.030	2.8%	1.419	0.051	3.6%	1.043	0.104	7.1%
Positive 3	0.891	0.036	4.0%	0.805	0.019	2.8%	0.769	0.041	5.3%	0.639	0.062	9.7%

**Table 3:** Number of swine herds and seroprevalence of SVA antibodies by province.

Province	^a^ Positive	^a^ Negative	^a^ Total	Ratio (%)
Henan	45	57	102	44.1%
Inner Mongolia	28	21	49	57.1%
Shanxi	12	3	15	80.0%
Fujian	4	3	7	57.1%
Shandong	3	4	7	42.9%
Sichuan	5	0	5	100.0%
Hubei	3	1	4	75.0%
Hebei	3	1	4	75.0%
Guangxi	3	1	4	75.0%
Jiangxi	2	2	4	50.0%
Guangdong	1	2	3	33.3%
Hunan	2	0	2	100.0%
Heilongjiang	1	1	2	50.0%
Gansu	1	1	2	50.0%
Anhui	1	0	1	100.0%
Zhejiang	1	0	1	100.0%
Jiangsu	1	0	1	100.0%
Shaanxi	1	0	1	100.0%
Yunnan	1	0	1	100.0%
Jilin	0	1	1	0.0%
Total	118	98	216	54.6%

Note: ^a^ represents the number of pig herds.

## Data Availability

The original contributions presented in this study are included in the article/Supplementary Materials. Further inquiries can be directed to the corresponding authors.

## Supplementary Materials

The following supporting information can be downloaded at: https://www.mdpi.com/article/10.3390/vetsci13010090/s1. Figure S1: Original images of SDS-PAGE and Western-blotting.

## Abbreviations

The following abbreviations are used in this manuscript:

ASFV	African swine fever virus
AUC	area under the curve
BSA	bovine serum albumin
CPE	cytopathic effect
CSFV	classical swine fever virus
CV	coefficient of variation
CI	confidence interval
DMEM	dulbecco’s modified eagle medium
*E. coli*	*Escherichia coli*
FBS	fetal bovine serum
FMDV	foot-and-mouth disease virus
IFA	immunofluorescence assay
IBRS-2	instituto biologico-rim suino-2
iELISA	indirect enzyme-linked immunosorbent assay
IPTG	isopropyl-β-D-1-thiogalactopyranoside
PRRSV	porcine reproductive and respiratory syndrome virus
P/N	positive-to-negative control
PCV2	porcine circovirus 2
PVDF	polyvinylidene fluoride
PCV3	porcine circovirus 3
PRV	pseudorabies virus
SVA	senecavirus A
S/P	sample-to-positive
SVDV	swine vesicular disease virus
TCID_50_	50% tissue culture infective dose
VNT	virus neutralization test
VSV	vesicular stomatitis virus
wpi	weeks post-immunization
