# Supplementary material for: Indirect ELISA Using Multi-Antigenic Dominants of VP1, VP2, and VP3 Recombinant Protein to Detect Antibodies Against Senecavirus A in Pigs
Source: Vet Sci. 2026 Jan 15;13(1):90. doi: 10.3390/vetsci13010090 (PMC12846615; doi:10.3390/vetsci13010090)
Supplement: Supplementary file 1 [file vetsci-13-00090-s001.zip › vetsci-4070342-supplementary.pptx]

## Slide 1
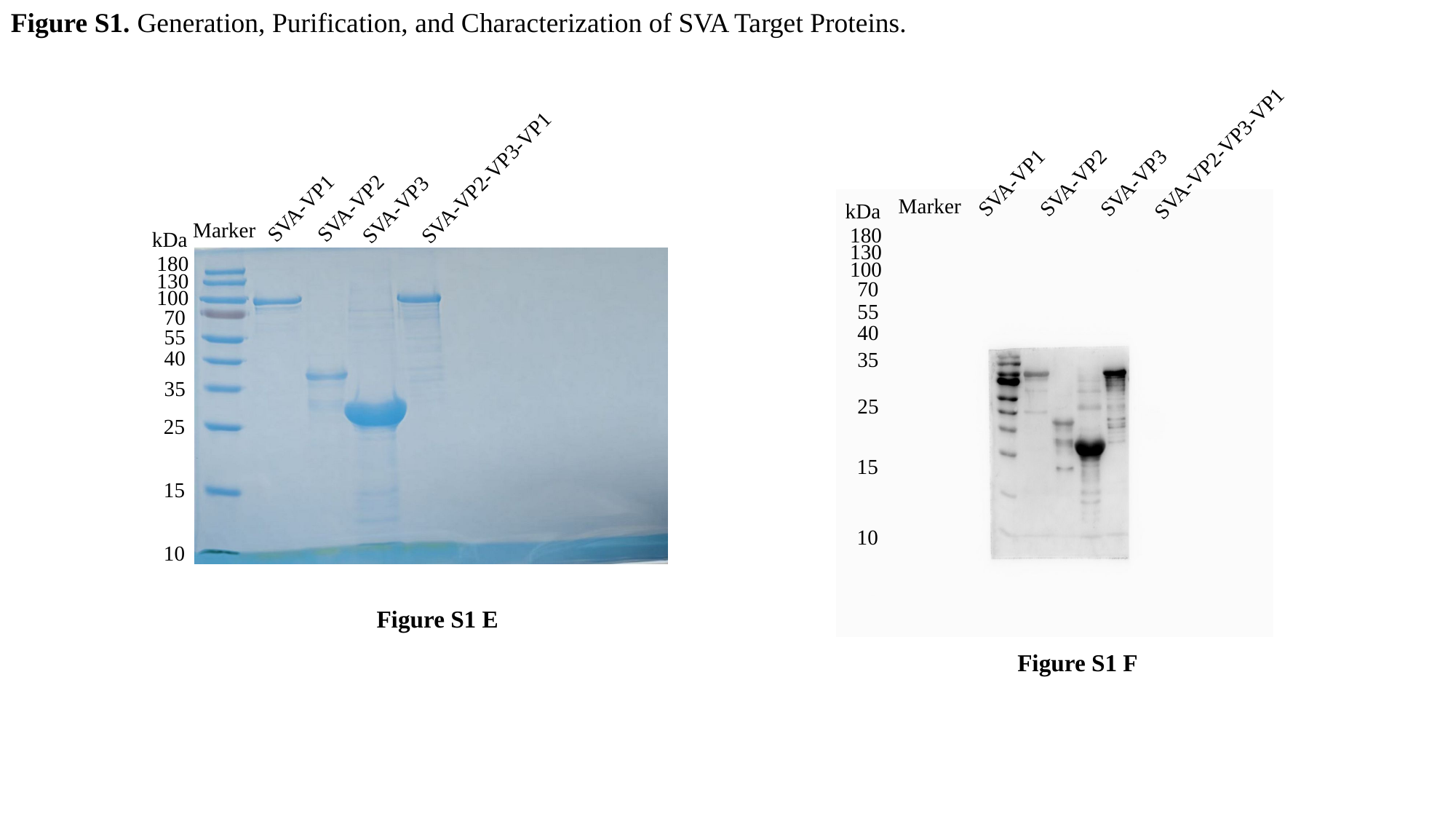

Figure S1. Generation, Purification, and Characterization of SVA Target Proteins.
SVA-VP2-VP3-VP1
SVA-VP3
SVA-VP1
SVA-VP2
SVA-VP2-VP3-VP1
SVA-VP1
SVA-VP2
SVA-VP3
Marker
kDa
Marker
180
kDa
130
180
100
130
70
100
55
70
40
55
40
35
35
25
25
15
15
10
10
Figure S1 E
Figure S1 F
